# Mule deer impede Pando’s recovery: Implications for aspen resilience from a single-genotype forest

**DOI:** 10.1371/journal.pone.0203619

**Published:** 2018-10-17

**Authors:** Paul C. Rogers, Darren J. McAvoy

**Affiliations:** 1 Western Aspen Alliance, Wildland Resources Department, and Ecology Center, Utah State University, Logan, Utah, United States of America; 2 Forestry Extension and Wildland Resources Department, Utah State University, Logan, Utah, United States of America; Austrian Federal Research Centre for Forests BFW, AUSTRIA

## Abstract

Aspen ecosystems (upland *Populus*-dominated forests) support diverse species assemblages in many parts of the northern hemisphere, yet are imperiled by common stressors. Extended drought, fire suppression, human development, and chronic herbivory serve to limit the sustainability of this keystone species. Here we assess conditions at a renowned quaking aspen (*Populus tremuloides*) grove—purportedly the largest living organism on earth—with ramifications for aspen biogeography globally. The “Pando” clone is 43 ha and estimated to contain 47,000 genetically identical aspen ramets. This iconic forest is threatened in particular by herbivory, and current management activities aim to reverse the potential for type conversion, likely to a non-forest state. We set out to gauge agents affecting recent deterioration through a network of monitoring plots and by examining a chronosequence of historic aerial photos to better understand the timing of putative departure from a sustainable course. Sixty-five permanent forest monitoring plots were located in three management regimes existing within Pando: no fencing, fencing with active and passive treatments, fencing with passive-only treatment. At each sample plot we measured live and dead mature trees, stem recruitment and regeneration, forest and shrub cover, browse level, and feces counts as a surrogate for ungulate presence. Ordination results indicate that aspen regeneration was the strongest indicator of overall forest conditions at Pando, and that mule deer (*Odocoileus hemionus*) presence strongly impacts successful regeneration. Additionally, fencing with active/passive treatments yielded the most robust regeneration levels; however, a fence penetrable by ungulates in the passive-only treatment most likely played a role in this outcome. The aerial photo sequence depicts various human intrusions over the past seven decades, but perhaps most telling, a decline in self-replacement beginning 30–40 years ago. Aspen communities in many locations in North American and Europe are impacted by unchecked herbivory. The Pando clone presents a unique opportunity for understanding browse mechanisms in a forest where tree genotype, closely aligned with growth and chemical defense, is uniform.

## Introduction

Aspen forests (chiefly *Populus tremuloides*, *P*. *tremula*) are among the most widespread tree systems in the world, yet their sustainability is threatened by human-induced impacts such as warming climates, development, fire suppression, and unchecked herbivory. These *Populus* systems support unusually high levels of biodiversity, when compared to surrounding conifer-dominated forests [1–6]. In aspen systems where rejuvenating fire does not play a large role [7], combined effects of herbivory and prolonged drought can be especially damaging to system resilience [8], as well as having cascading effects on biodiversity [9]. In a North American management context, these elements—forestry, wildlife, disturbance ecology—are often addressed within distinct disciplinary “silos.” This situation constitutes a challenge for sustainable stewardship in the face of expected near-term climate warming and altered precipitation patterns leading to extended seasonal droughts [10].

Increased ungulate pressure on key forest species is brought on by reductions in apex carnivores, as well as policies encouraged by sportsmen to maintain high game populations. Within the context of North American aspen, single-species game management has potentially catastrophic effects on a much wider range of plant and animal species which thrive where aspen are intact. Seager et al.[11] addressed this issue in-depth and recommended that herbivory monitoring for both wild and domestic species be prioritized. While reintroduced predators appear to have positive effects on mediating chronic aspen browse problems[12], further work is needed to fully understand how ‘trophic cascades’ play out under varying aspen functional types[13,14]. Plus, studies addressing predator-herbivore-aspen relations have been confined to only a few landscapes where gray wolves (*Canis lupus*) have been reintroduced. This is a tiny portion of the greater western aspen range. Additionally, much of the research focus across the western U.S. addressed Rocky Mountain Elk (*Cervus elaphus*) browsing, with less energy examining long-term impacts of mule deer (*Odocoileus hemionus*) on aspen systems [11]. Where these species co-exist, often alongside domestic cattle (*Bos* spp.) or sheep (*Ovis* spp.), elk seem to have greater impacts [15–16], perhaps because their dietary needs are seasonally more flexible than other regional ungulates [17]. When focusing on only deer and domestic cattle, both species favor browsing aspen suckers late in the season when other forage senesces, although deer will generally avoid cattle when the two comingle [18].

The Pando aspen clone is putatively the largest known organism on earth in terms of dry-weight mass [19]. This unique ‘forest of one tree’ was first described in the 1970s, in part, as an exercise in determining clonal differences based on leaf morphology and other traits [20]. The clone was later named “Pando” (Latin: *I spread*) based on its vegetative reproductive strategy and alleged ancient lineage [19]. More recent work in molecular ecology confirmed the original size estimate of 43 ha [21] and, although clone age cannot be specifically determined at this time, a gross estimate of a post-glaciation origin seems plausible [22], meaning the clone is likely thousands of years old though not older than 14,000 years. Recent degradation of Pando had been noted by area managers, which instigated a formal study to understand mechanisms at work by conducting restoration experiments within a limited area of the clone [23]. Results from this work suggested that forest treatments of burning, shrub removal, and partial cutting were effective stimulants of vegetative suckering, but that fencing alone (i.e., protection from herbivores) produced more than enough ramets to replace dying mature stems. What is missing, however, is a comprehensive assessment of the entire clone, including a newly fenced larger section of Pando, in order to understand how the different management regimes are playing out in this renowned grove.

The purpose of this study is to understand the key dynamics that affect the sustainability of this forest and how its unique character may inform threatened aspen communities at-large. In order to do this, we undertook a baseline assessment of the entire Pando clone with the goal of documenting its status so that we can further track changes in the clone based on restoration actions and follow-up monitoring. Specifically, this research has three aims: 1) to present a baseline description of current conditions at the Pando aspen grove; 2) to utilize a *de facto* experimental design—No Fence, 2013 Fence (active/passive treatments), and 2014 Fence (passive treatment only)—to understand causal agents influencing Pando’s current state; and 3) address restoration implications for Pando and relate these to aspen conservation and management at continental scales. Aspen communities in North America and Europe are being affected to varying degrees by chronic herbivory [3,8–9,11]. Fully understanding mechanisms at work in a forest without genetic variation presents a unique opportunity to control for traits such as growth and chemical defense, which are closely linked to genotype. Lessons learned here, where genetic properties have favored Pando’s long-term persistence, may be used to course-correct locally, as well as provide direction for widespread aspen conservation. If we can better understand aspen dynamics and restoration in a semi-controlled environment where an entire forest is a single genotype, then this will have implications (e.g., growth vs. defense ecological strategies; population and movement herbivore management) for wider aspen conservation questions where herbivory plays a central role. Extrapolation of results from Pando carries limitations, too; though herbivore-plant impacts to an entire forest of one genotype allows us to control for genetically-driven traits (defense, disease susceptibility, growth potential, etc.) this unique feature is less helpful in informing responses to environmental impacts, such as prolonged drought or wildfire.

## Methods

### Study site

The Pando aspen clone is located in south-central Utah on the Fishlake National Forest (UTM 434701 E, 4264266 N). The average elevation is 2,707 m, with slopes ranging from 5–10 percent and generally SE trending. Soils are mixed gravelly and cobbly in both loamy A-horizons and clayey B-horizons (Draft Survey, Fishlake National Forest, National Cooperative Soil Survey UT651) originating from tertiary volcanic materials—likely basalt, rhyolite, and latite welded tuff (*Personal comm*., Mike Domeier, NRCS Utah). The forest floor is sparsely vegetated, with intermittent exposed volcanic boulders and common bare ground patches. Common juniper (*Juniperus communis*) and mountain big sagebrush (*Artemisia tridentata*) are densely clumped throughout the site. Annual precipitation is attributed mostly to winter snow, though a secondary surge of rainfall occurs in July-August (466 mm^yr-1^ precipitation, SNOTEL 1149, 6 yr. average).

Pando is bisected by a paved state highway accessing a popular resort area and bordered by a public campground, as well as private summer homes. Thus, human presence is nearly constant in the summer, with less traffic the remainder of the year. Small clearfell-coppice cuts were performed near the edge of the clone in 1987 and 1988, but were unfenced. An additional cut took place in 1992, though managers at that time elected to fence this treatment due to heavy browsing following the earlier cuts. Currently, domestic cattle (*Bos spp*.) forage at Pando under a U.S. Forest Service grazing allotment for approximately two weeks annually. Mule deer (*Odocoileus hemionus*) and Rocky Mountain elk (*Cervus elaphus*) access this area freely during the typically seven month snow-free season. (Elk sign is evident in the broader area, although we have not seen elk on site nor found scat in Pando surveys.) Most human visitors are unaware of the significance of this unique forest, but efforts have commenced to provide greater public awareness and education regarding Pando. While citizens are commonly in the area during summer months, field efforts aim to avoid attracting public attention which may otherwise draw interest toward sample locations, potentially resulting in permanent marker removal, increased trampling, or undue vegetation damage.

### Field sampling

Our sampling design and methods were adopted from an earlier study [23] and expanded to characterize the entire 43 ha Pando aspen clone. Data collection took place during June of 2016 and 2017. The experimental design consists of three distinct management regimes that have evolved over time and were not purposely intended as “treatments” in the traditional sense. This non-traditional, post-hoc, approach should therefore be considered exploratory in nature with the central objective being the understanding of how management regimes may have resulted in varying resilience outcomes. The first treatment is the “No Fence” zone which, as the name implies, is completely unprotected from browsing ungulates. There has been no active management of the forest in this zone in recent years.

The “2013 Fence” area contains three experimental treatments (burning, shrub removal, selective tree cutting) with significant passive treatment (fence only), as well. Shrub removal and tree cutting were conducted by U.S. Forest Service work crews using hand tools and did not involve heavy equipment. Fence construction did, however, utilize a small treaded tractor which likely involved localized soil and root damage. A previous study [23] details spatial alignment of active treatments, as well as aspen response to treatments. In brief, that work found that equal areas of burning, cutting, and shrub removal resulted in no differences in regeneration between activity types three years after treatment. There was a significant difference between all treatments grouped and no-treatment regarding regeneration response when all were protected from herbivory, however the entire 2013 fenced area (i.e., treatment and no-treatment) produced adequate regeneration for overstory replacement [23].

**Fig 1:**
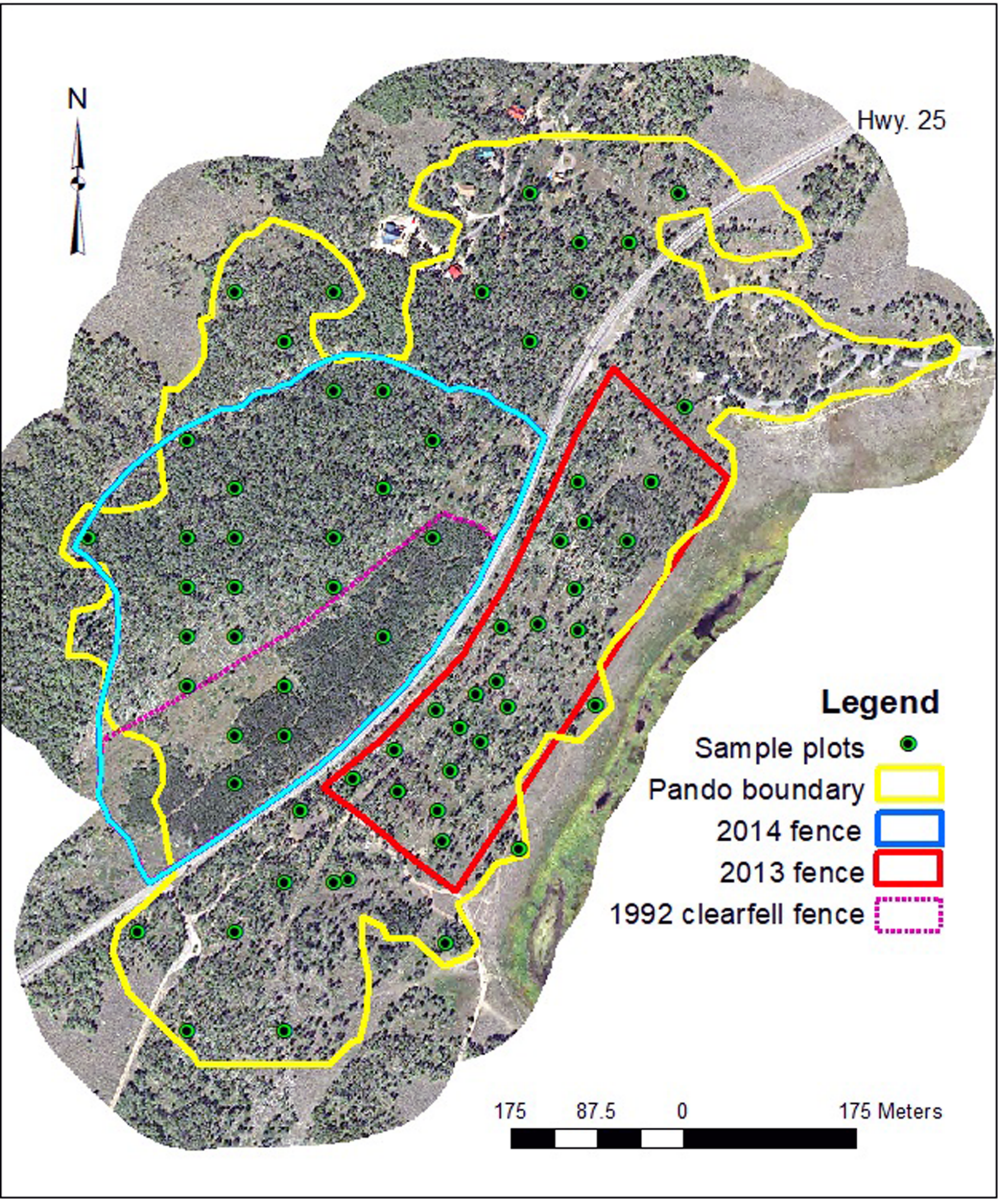
Map depicting the study area within the Pando aspen clone, Utah, USA. Sixty-five sample plots were randomly distributed across the study area, with equal portions located within No Fence, 2013 Fence, and 2014 Fence management regimes. Base image courtesy of USDA Aerial Photography Field Office.

The third treatment area is the “2014 Fence” which was larger than the earlier fenced area and contained no active treatments. We did not consider the 1992 clearfell-coppice within the 2014 Fence as active treatment because of the 25-year interval that has elapsed since that activity and an apparent lack of effect this disturbance is having on contemporary aspen regeneration. This approach was taken largely based on the initial regeneration success of the 2013 fence. Significantly, as a cost saving measure, the 2014 fence construction used a portion of a 22-year-old fence originally designed to protect a clearfell-coppice (Fig 1).

Individual plots constitute the primary sample units of this work. All plots are located within the Pando clone itself; meaning that the entire study measures tree and forest conditions within a genetically uniform triploid aspen clone [21,24]. Plots were chosen via a random sub-selection from a 50 x 50 m base grid. Tree- and browse-centered data elements were recorded within two 30 x 2 m transects arranged in a perpendicular fashion to avoid potential slope biases. For our purposes, the fixed area within transects is synonymous with the “plot” and is assumed to characterize conditions across that portion of the treatment area. This method allows easy expansion of values to a ha^-1^ basis for analysis (i.e., S1 Table). The three treatments were No Fence (~21 ha), 2013 Fence (~7 ha), and 2014 Fence (~15 ha) as described above. Greater plot density was required within the 2013 Fence in order to achieve a relatively uniform sample size (plots) for each treatment (Fig 1). Approximately two-thirds of the 2013 fenced area was not actively treated and we sampled both active (8 plots) and passive (13 plots) areas to account for those proportions. In sum, there are 65 sample locations in this study.

At each sample plot we recorded data elements characterizing site, forest/tree, browse, and herbivore conditions. Environmental variables describing each site (plot) included geographic position (UTM), elevation, number of stand layers (distinct vegetation height layers), stand condition (a qualitative score validated previously [8]), percent aspen cover (% sky blocked by aspen trees within 2 m radius looking upward; 14 observations averaged), treatment type, juniper cover (averaged two transect-based estimates), and a comments section for describing unique conditions not covered by other measures. Due to nearly uniform low values across the study area for the number of layers and stand condition ratings [8] we did not conduct further analysis on these elements, but recognized their value in documenting baseline conditions for future study. Actual sampling began by photo documenting transects at each end facing toward the plot center, thus archiving a record of repeat photography in the four cardinal directions along measurement transects upon each visit. For all trees within transects ≥ 8 cm diameter at breast height (dbh) we recorded tree species, status (live/dead), and tree diameter class (≥ 8–15, > 15–25, >25 cm). A count of aspen regeneration (i.e., stems ≤ 2 m height) were made within three height classes (0–0.5, > 0.5–1, > 1–2 m). Missing terminal buds determined to be due to browsing were recorded for each regenerating ramet. Live aspen recruitment (i.e., stems > 2 m height, < 8 cm dbh) was also tallied within sample transects. Stems were considered separate ramets if they forked below the soil litter. Finally, we counted domestic and wildlife herbivore scat presence within sample transects [8]. Domestic livestock feces were tallied per individual deposit. For wild ungulates, “piles” were considered separate if distinct groupings of pellets included at least three pellets [25]. Scat piles were only counted one time per sample location. After counting, piles were removed from transects to ensure that remeasurements would only tally new scat. Scat presence on plots within any fenced area is indicative of some degree of unsuccessful exclosure.

### Aerial photo chronosequence

**Fig 2:**
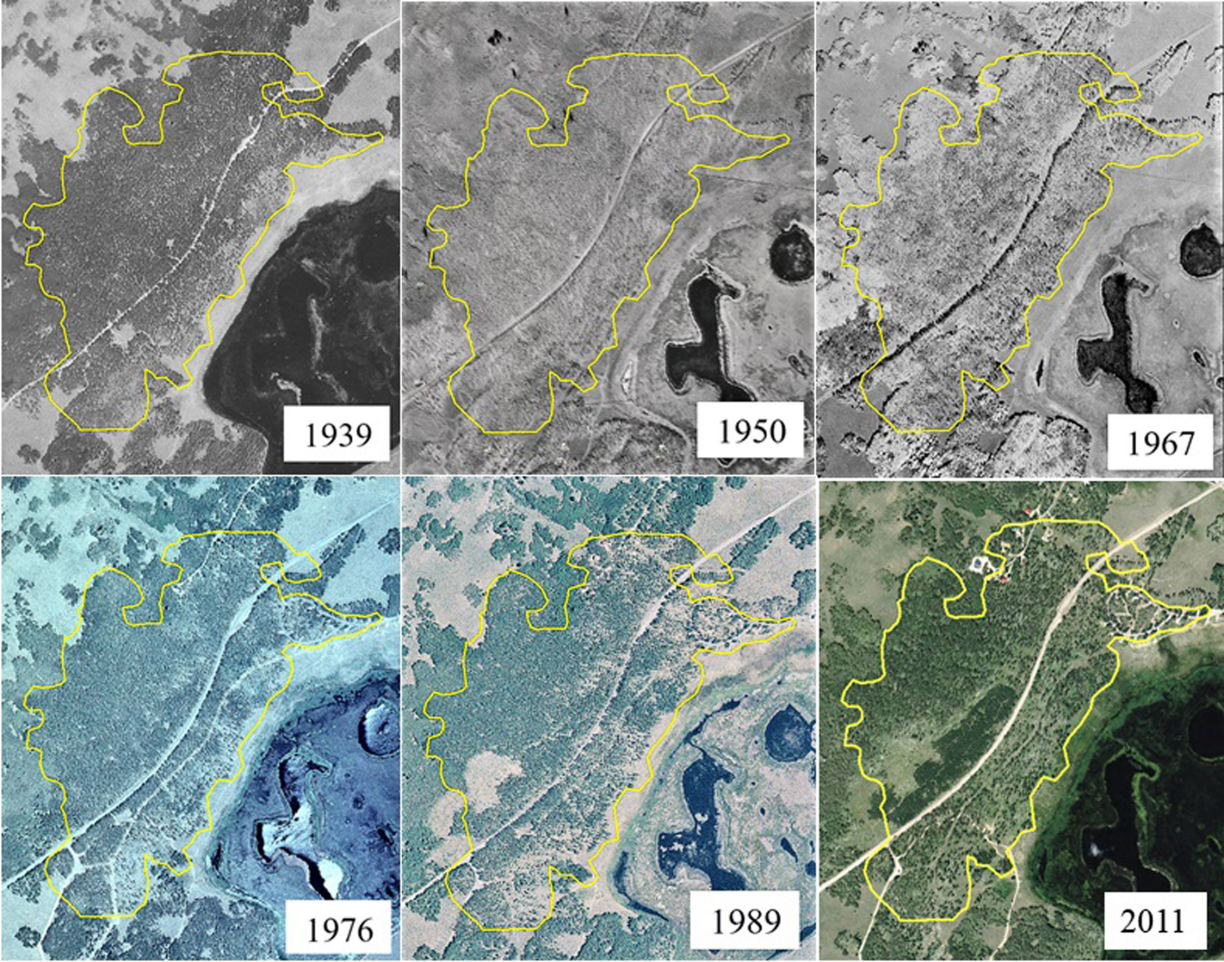
A seventy-two year aerial photo chronosequence showing forest cover change within the Pando aspen clone, Utah, USA. Photos were georectified using ArcMap^®^ software to ensure accurate scale and location alignment. Yellow polygon depicts the boundary of the 43 ha clone as projected over each photo year. Base image courtesy of USDA Aerial Photography Field Office.

In undertaking a complete measure of Pando’s current status, we felt it important to gain insight into the recent past by systematically viewing aerial photography of the grove. Our hope was that this information would complement on-the-ground data collection by presenting a multi-decadal time sequence of forest stand change. In searching online and U.S. government digital aerial photo repositories we were able to build a catalogue of nine photo coverage years at Pando, six of which provided ample clarity for interpretation. These photo years (1939, 1950, 1967, 1976, 1989, 2011) were then digitally rectified using ArcMap^®^ v.10 software so that each could be directly compared at the same scale. All photos used were from public sources. Separately, we used the genetic sampling work of Mock et al. [21] to digitize an accurate Pando clone boundary. Placement of this boundary, also georectified, over each photo provides a consistent frame for viewing changing forest conditions over this 72 year time period (Fig 2).

### Analytical methods

In order to understand conditions governing Pando’s various management regimes our analytical approach strove to first use the entire “data landscape” in determining the most useful variables, second to examine the degree of group solidarity using all measures, and third to use the highest performing indicators to test actual group differences. We used PC-ORD^®^ v. 7.0 software [26] for statistical analyses in the first two steps, then the SAS^®^ statistical package (SAS Institute Inc.) to test group differences. For exploratory analyses of all data elements and dimensions we used nonmetric multi-dimensional scaling (NMS) to evaluate performance of individual variables in describing conditions at Pando. NMS is an ordination process well suited for ecological questions where multiple data types are used, common parametric standards are not always met, and data sets may contain large numbers of zeros [27–28]. In exploratory mode, NMS helps to reduce complex multidimensional data sets to a few key explanatory gradients (axes). Our primary matrix for this analysis consists of all plots (rows) pitted against all sampling variables (columns). A secondary matrix of environmental variables (removing categorical variables) by plots was evaluated in relation to the main variable ordination for display purposes. The lowest stress solution was derived from 250 runs with real plot data. “Stress” is a quantitative assessment final NMS solution monotonicity, a measure of how well real data fit the ordination [27,29]. The lowest stress solution was subjected to a Monte Carlo test of an additional 250 randomized iterations to evaluate the probability of the final NMS solution being greater than chance occurrence (i.e., provides a *p*-value). Orthogonal rotation of the final ordination was used to maximize correlations between the strongest environmental variables (i.e., Pearson r values) and the major ordination axes. The lowest number of dimensions (axes) was selected when adding another dimension would have decreased the final stress by <5 [27].

Our second analytical step was designed to understand if there were differences between our broad treatment groups at Pando. Multi-Response Permutation Procedures (MRPP) is a nonparametric test for describing within group agreement of variables in contrast to adjoining data groups. We selected MRPP using the Sørensen distance measure because it is less inclined to exaggeration based on outliers and zero values [29]. All categorical variables were removed from the data matrix except for our group designator, thus this analysis was performed on a matrix of 65 stands by 13 variables. MRPP produces a T score indicating the degree of difference between group pairs, an A-value which is the chance-corrected within group agreement (effect size), as well as a *p*-value establishing level of test significance [27].

After testing variable and group differentiation with the aforementioned procedures, our final examination used two standard non-parametric procedures to assess differences in key metrics between the three treatment types (No Fence, 2013 Fence, and 2014 Fence). This simultaneous 3-way group test employed the Kruskal–Wallis test, a non-parametric equivalent to analysis of variance. Where results suggest two similar groups with a third group driving the significance of difference, we further tested those similar groups using a 2-sided Wilcoxon–Mann–Whitney *U* test. Output from both of these tests are reported in mean Wilcoxon scores. Results are considered significant where a Monte Carlo-simulated chi-square test using 10,000 runs produces a significant *p*-value [30]. For all tests we used a 95% confidence level (*p* ≤ 0.05) to determine significance.

## Results

### Baseline assessment

A qualitative examination of a 72-year aerial photo sequence of the Pando aspen clone revealed distinct changes in forest cover during this period (Fig 2). Other than the road alignment through the study area, which remains constant throughout, few of the forest openings found at the end of the sequence (2011) are evident in the 1939 photo. Other prominent trends include a general thinning of the forest downhill (right of) the road, the addition of a campground and recreational homes (upper right and left, respectively), and silviculture treatments above the road in the center-left portion of the photos as depicted in the final two photos (1989, 2011). Overall, the photo sequence illustrates a reduction in aspen cover during this period.

To fully understand variation in forest metrics within the present day Pando clone we compiled baseline statistics by broad treatment categories (Table 1). Standard deviations for most variables are large due to common occurrence of wide variances and zero values in the data set. Cover of mature aspen, as well as the understory shrub *Juniperus communis*, was slightly higher within the 2014 fenced area than the other two zones. Aspen regeneration is notably higher in the 2013 fenced area (examined in-depth below), though within group variance (SD) is high. Browse levels are non-existent post-fencing inside the 2013 fence, but are moderately high in the 2014 fence and where there is no browsing protection. In the 2013 fenced zone, mature tree counts per ha^-1^ were lower, while live and dead basal area volume were greater than the other groups. The lowest percent standing dead basal area falls within the 2014 fenced area, though it should be noted that 24–37 percent standing dead tree volume across treatment types (many more are on the ground, but not tallied here) suggests a high mortality rate, overall.

**Table 1:** Summary statistics for all locations by treatment group. All values represent group means (SD), except for number of plots. Regeneration are young aspen stems ≤ 2 m in height. Percent browse measures are only taken from regeneration stems. Recruitment are stems > 2m in height and < 8 cm diameter at breast height.

Treatment	Number sample plots	Percent juniper cover	Percent aspen cover	Aspen regeneration ha^-1^	Percent browse	Aspen recruitment ha^-1^	Live trees ha^-1^	Basal area live trees m^2^ ha^-1^	Dead BA as percent of total BA
No Fence	22	22(21)	23(11)	299(683)	55(47)	16(35)	416(400)	13(16)	30(44)
2013 Fence (Treatment-No Treatment)	21	24(17)	17(8)	1698(1078)	0(2)	60(70)	325(199)	21(20)	37(56)
2014 Fence (No Treatment)	22	35(16)	28(13)	151(266)	24(36)	1204(2261)	470(418)	18(21)	24(30)

### Seeking explanatory variables of Pando’s status

**Fig 3:**
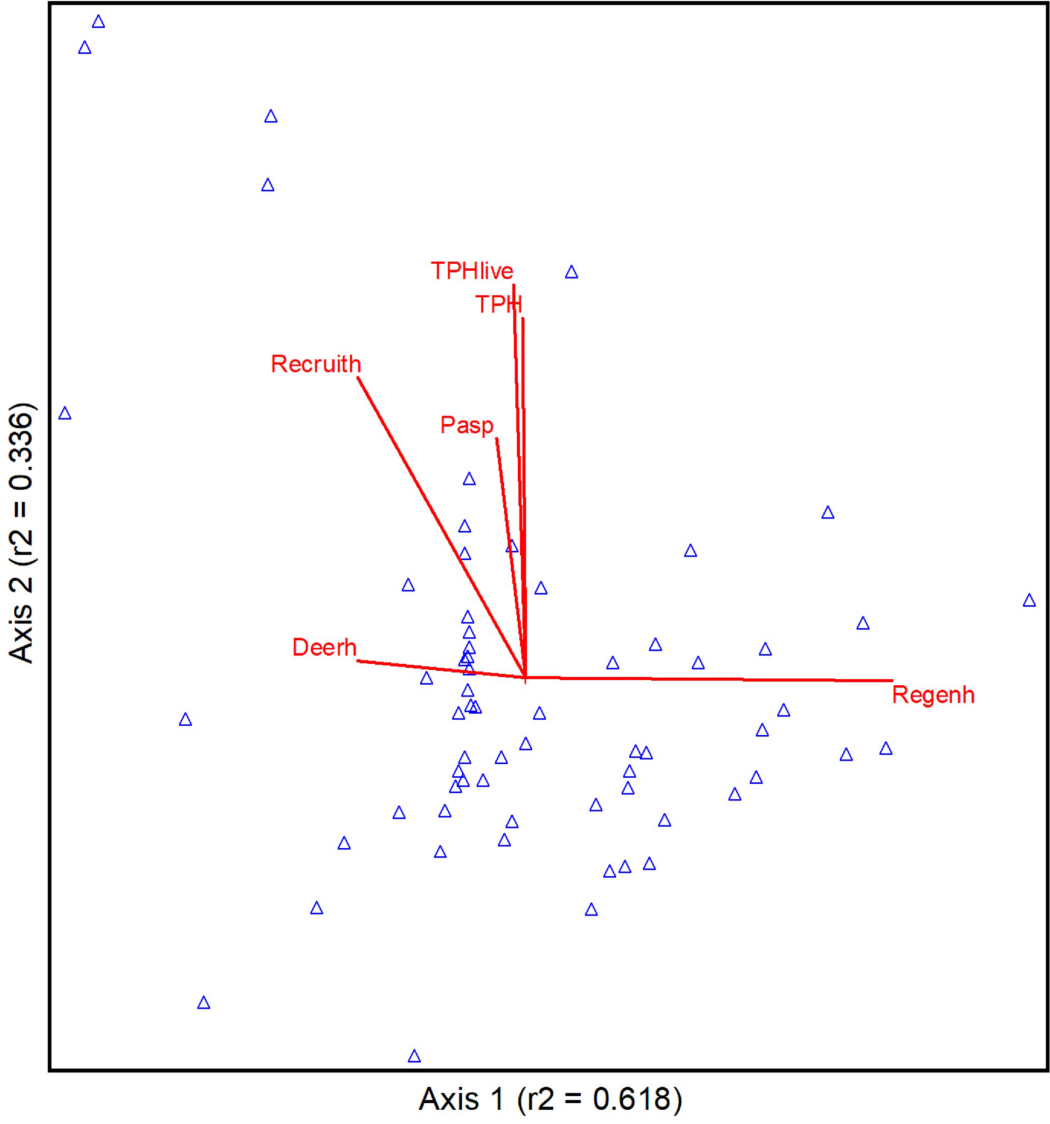
A joint plot depicts the results of nonmetric multidimensional scaling (NMS) ordination on a matrix of 65 plots by 16 monitoring variables. Highly correlated environmental variables (*r*^2^ ≥ 0.3) are overlaid on the ordination to show relationships to primary axes. Vectors indicate direction and strength (length) of these factors in the ordination space defined by plot values of all measured variables. Variables shown are: deer pellet groups ha^-1^ (Deerh), aspen recruitment stems ha^-1^ (Recruith), percent aspen cover (Pasp), live mature trees ha^-1^ (TPHlive), trees ha^-1^ (TPH), and aspen regeneration stems ha^-1^ (Regenh). Triangles show locations of plot scores in “data space” within the NMS ordination.

Ordination analysis resulted in a two-dimensional solution on a matrix of 65 stands by 16 variables. We projected the results of the NMS in ordination “plot-data space” as a joint plot using a subset of 12 environmental variables, where the most highly correlated (*r*^2^ ≥ 0.3) results are displayed as an overlay (Fig 3). The final NMS solution produced a stress value of 9.06 with an instability of 0.00. A Monte Carlo test of 250 random data runs versus the real data set verified a significant NMS outcome (*p* = 0.004). The two-axis solution described about 95% of ordination variance (axis 1: r^2^ = 0.618; axis 2: r^2^ = 0.336; orthogonality = 90.0). Length and direction of vectors corresponds to environmental (explanatory) variable strength and relationship to the two-dimensional plot-data space. Table 2 presents NMS results by axes for all environmental variables. Most measures tested in this ordination contributed very little to environmental gradients (NMS axes) at Pando. Strong (*r*^2^ ≥ 0.3) positive and negative responses to axis 1 (Fig 3) represent factors working in opposition in terms of their influence on the totality of plot variable values at Pando. As the stronger of the two dimensions represented here, axis 1 describes a gradient of regeneration abundance with a negative correlation to deer presence. Axis 2 displays no negative elements, though a positive gradient is evident in our data set with mature aspen tree counts and tree cover. Basal area also correlated positively with axis 2 (Table 2), but was just below our criteria (*r*^2^ ≥ 0.3) for joint plot display. A moderately strong correlation of aspen recruitment to both axes does not present a clear signal, but is likely related to overall low recruitment at this time and spatially isolated high recruitment in the historic (1992) clearfell-coppice treatment (Fig 1, violet dotted line).

**Table 2:** Pearson's coefficients (*r*) between environmental variables and primary NMS ordination axes. The strongest response variables are highlighted in bold type, where *r* > 0.5 or < -0.5.

	*r—*Value	
Variable Name	Axis 1	Axis 2
Treatment	-0.275	0.311
Fence	-0.225	0.252
Condition	-0.244	-0.061
Elevation	-0.343	0.108
Juniper Cover	-0.095	0.210
**Aspen Cover**	-0.237	**0.680**
**Regeneration ha**^**-1**^	**0.841**	-0.083
Browse Level	-0.144	-0.155
**Recruitment ha**^**-1**^	**-0.570**	**0.761**
**Trees ha**^**-1**^	-0.076	**0.833**
**Live Trees ha**^**-1**^	-0.150	**0.872**
BA Live	0.083	0.436
BA Dead	0.068	0.063
BA Total	0.090	0.389
Cattle	-0.200	-0.211
**Deer**	**-0.570**	0.183

### Results for group differences

The Multi-response Permutation Procedures (MRPP) results show that there is solid alignment, based on 13 plot variables, of within group agreement (i.e. group validation); essentially confirming that values within the treatment groups are more similar than those between groups (Table 3). Although all comparisons were highly significant, greater negative T values indicate bigger differences between groups. The markedly smaller negative T value in the No Fence *vs*. 2014 Fence result indicates more agreement (less separation) in these groups. After establishing statistical distinctions within groups based on multivariate analysis, we then examined treatment differences based on our strongest single indicator (regeneration) variable from the dominant explanatory axis of the NMS ordination (Fig 3).

**Table 3:** Multi-Response Permutation Procedures (MRPP) test results for differences in cumulative scores for all variables between treatment groups. “T” is the MRPP test statistic which calculates the difference between observed and expected delta. “A” is the chance-corrected within-group agreement.

Treatment Group	T	A	*p*
No Fence *vs*. 2013 Fence	-14.08	0.17	<0.001
No Fence *vs*. 2014 Fence	-4.17	0.06	0.003
2013 Fence *vs*. 2014 Fence	-16.3	0.20	<0.001

**Fig 4:**
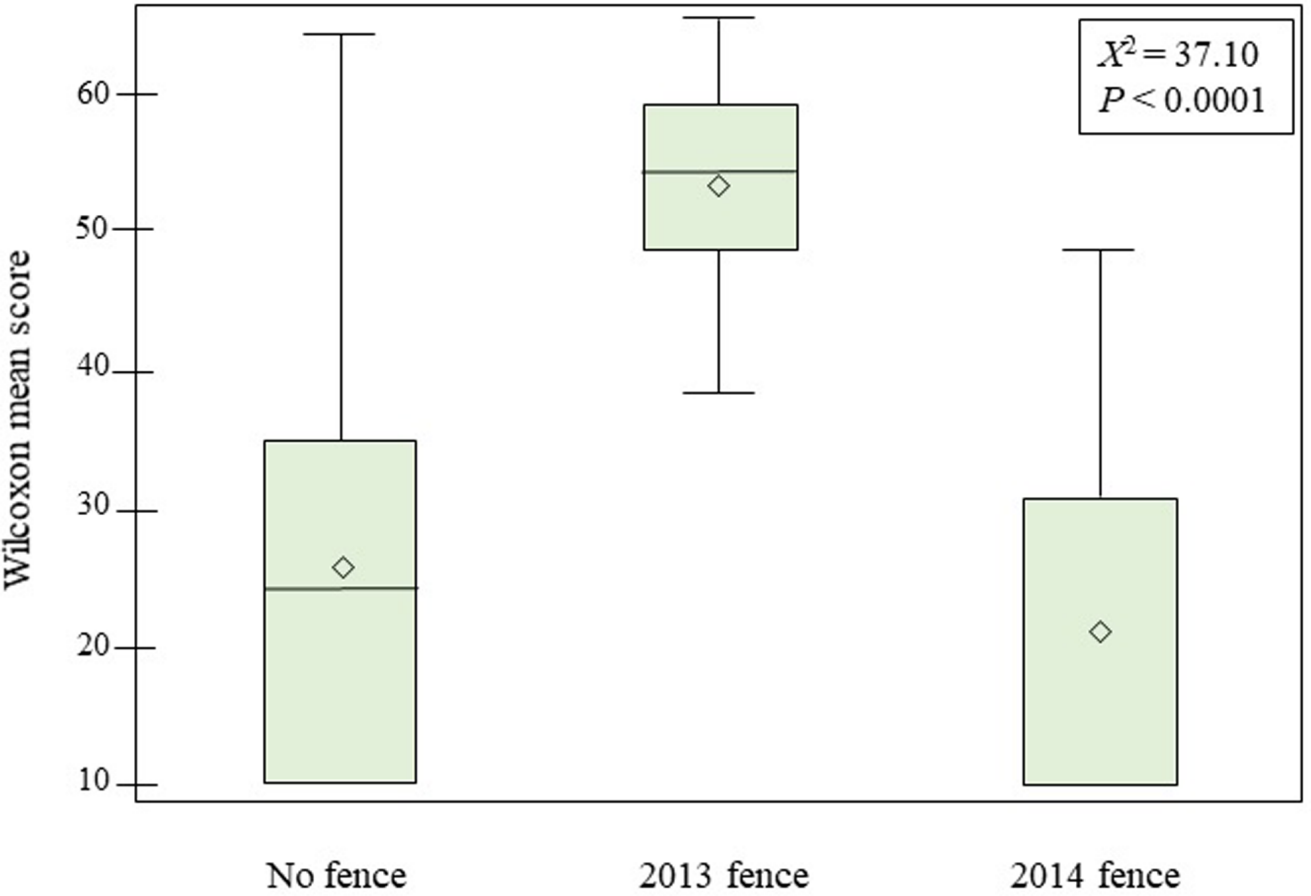
Box plots depicting a significant difference (χ^2^ = 37.10, *p* < 0.0001) in terms of the Kruskal–Wallis test for differences between treatments (groups) of regeneration ha^-1^. Output from Kruskal–Wallis test is shown in Wilcoxon mean scores on the y-axis (SAS^®^). Whiskers show minimum and maximum values, boxes represent 25–75% data ranges, horizontal lines within boxes are medians (no line indicates Wilcoxon score of zero), and diamond symbols are means. Results are considered significant where a Monte Carlo-simulated chi-square test using 10,000 runs produced an estimated *p*-value of <0.05.

**Fig 5:**
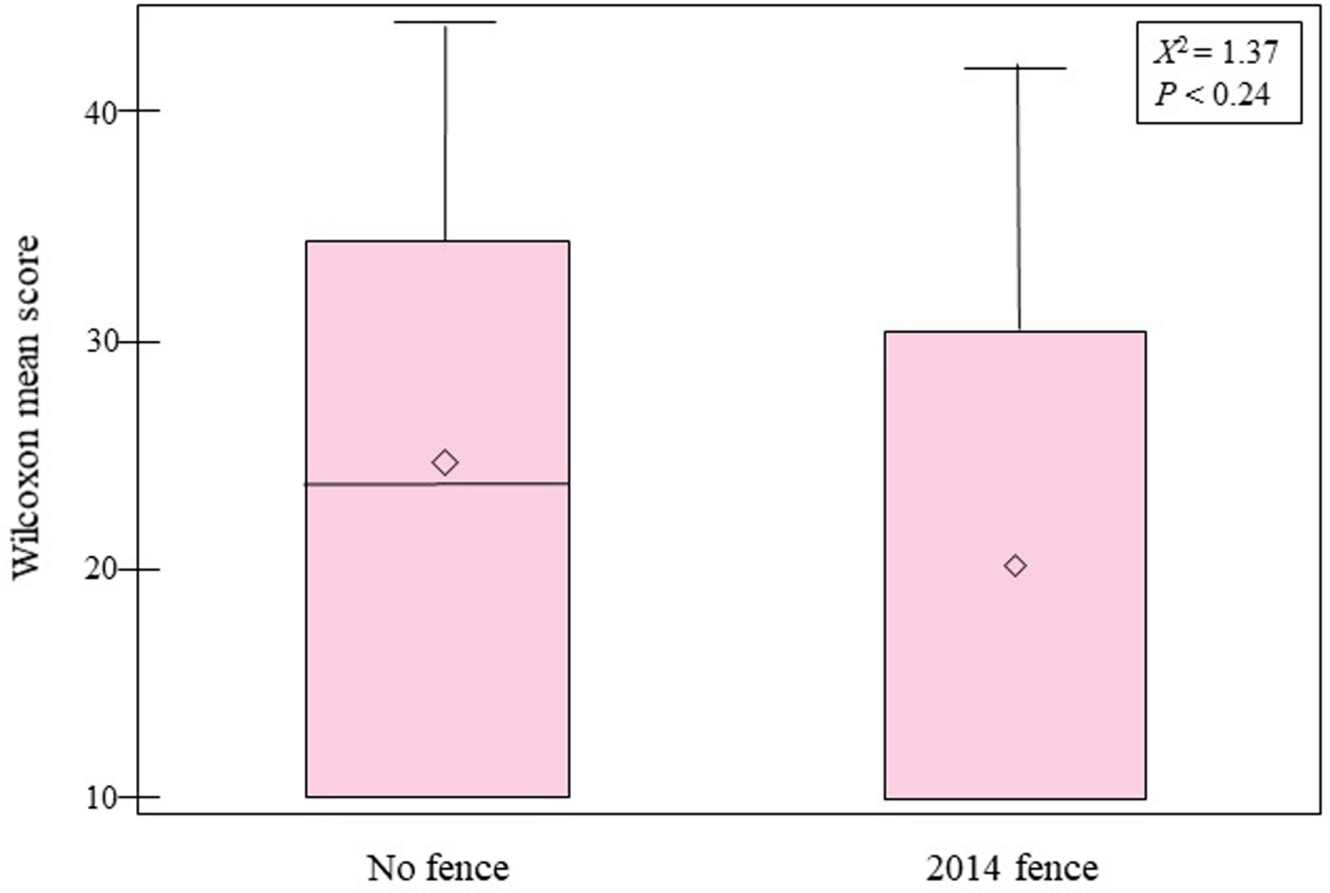
Wilcoxon–Mann–Whitney *U* test results displayed in box plots showing no significant difference (χ^2^ = 1.37, *p* < 0.24) in regeneration ha^-1^ between No Fence and 2014 Fence treatment groups. Output from the test is shown in Wilcoxon mean scores on the y-axis (SAS^®^). Whiskers show minimum and maximum values (no line indicates Wilcoxon score of zero), boxes represent 25–75% data ranges, horizontal lines within boxes are medians, and diamond symbols are means.

The non-parametric Kruskal-Wallace test for treatment effect based on regeneration response yielded a significant difference among groups (χ^2^ = 37.10, *p* < 0.0001). Most of that difference appears to come from the strong regeneration response of treatments and protection within the 2013 Fence group (Fig 4). A two-way comparison between the remaining treatment groups resulted in no significant difference in regeneration response (Mann-Whitney-*U* test; χ^2^ = 1.37, *p* < 0.24), suggesting little positive effect from 2014 fencing to protect from browsing deer and cattle (Fig 5).

## Discussion

### Status of an ancient large aspen clone

We undertook a study of a 43 ha aspen clone using multiple lines of inquiry to determine its recent past and current status. The Pando aspen clone has likely survived millennia [22] at its present location, but it is clearly losing ground in the past half-century. Evidence from seven decades of an historical photo sequence (Fig 2) alongside the first comprehensive survey of forest conditions at Pando depict a deteriorating situation with the exception of the experimental fencing that was erected in 2013 (Fig 4). A detailed study [23] conducted earlier centered in and around the 2013 exclosure showed that both treatment and no-treatment provided adequate regeneration; elimination of browsers via fencing was the key component of regeneration success. Rogers and Gale [22] found no significant difference between treatment types regarding regeneration response. The area encompassed by the 2013 fence amounts to approximately 14% of the total clone. The remaining fenced and unfenced portions of Pando continue to display steady mortality in the overstory and virtually no successful reproduction in the understory or recruitment to intermediate stages (Table 1 and Fig 4). The 1992 clearfell-coppice cut (discussed below) provided all of the “recruitment” within the 2014 fence (thus, high mean and standard deviation; Table 1), with no successful recruitment since that time.

From 1939 to 2011 the signs of human intrusion become steadily more evident (Fig 2). While we would expect mature trees to be impacted by development, forest clearing, and stand aging, closer visual inspection of this photo chronosequence reveals consistent lack of regrowth (i.e., increased inter-tree spacing) from the 1970s to the present. Previous work attests to the importance of continual regeneration and recruitment in stable aspen types [13,31–32], such as the Pando clone. Where seral aspen communities are most often regenerated by stand-replacing events, stable aspen reproduce via individual tree and small gap replacement. The regenerative strategy of this functional type, therefore, is highly dependent on a complex vertical forest structure composed almost entirely of aspen with little or no competition from other tree species [13]. The clearfell-coppice cuts performed in 1987–88 (lower left, straddling clone boundary) have not reproduced any forest cover since. The 1992 tree removal and subsequent fencing was “successful” in that a cohort of recruitment exists today (Fig 1), although the uniform tree height resulting from a stand-replacing event poorly mimics (at least short-term) stable aspen stand architecture. It should also be pointed out that the 1992 fence provided enough short-term protection for (now) sapling-sized aspen to survive, though nearly all new suckers in this area are being browsed. Given no change in current practices, it will take several decades for this disturbance type to return to a stable, uneven, stand structure as would be expected had the clearfell and chronic browsing not occurred.

### The role of herbivory in a single-organism forest

Results from this assessment of Pando’s current condition strongly suggest that herbivory is playing a central role in the deterioration of this iconic grove. Whereas previous work showed that protection from herbivory alone produced sufficient replacement ramets for the dying mature stems [23], the current study indicates there is a clear inverse correlation between deer presence and regenerating aspen suckers (Fig 3). A stronger correlation to axis 1 in browse level (Table 2) would have provided a greater indication of causality. We believe the small amount of total regeneration (i.e., small sample; many zero counts) within the 2014 Fence and the No Fence groups led to an indefinite ordination result. The lack of differentiation between the 2014 Fence and No Fence groups (Fig 4) is a setback to forest managers who invested heavily in the fencing. Our recorded browse rate within the 2014 Fence of 24% corroborates past research suggesting that aspen sucker browse rates > 20% should be considered non-sustainable over time [33–34]. Poor performance in excluding browsers must be attributed to penetration of the fence by mule deer, as numerous sightings and photographs of deer within this exclosure have been noted. We speculate that the “weak link” in the 2014 fence, at least in part, is due to incorporation of older (1992) fencing that deer appear to exploit as entry ways (e.g., gaps between panels, under the fence, or low-hanging panels all due to fence age).

What makes Pando a unique laboratory for forest ecology experimentation and restoration is, of course, it existence as a ‘forest of one tree’ [20–21]. In relation to herbivory, we know chemical defense in the form of phenolic glycosides can be an effective ungulate deterrent and its allocation is aligned with genotype [35–36]. And though we are aware that defense chemistry, even within clones, varies with ramet age there is solid evidence to suggest that all young suckers would have similar phenolic glycoside levels [37].Thus, a single, albeit very large aspen genet, would presumably carry the same level of chemical resistance to herbivory across its 43 ha area. In its current state, Pando appears to exhibit a relatively low rate of chemical resistance, although this conjecture has not been tested. We may presume, however, that this long-lived very large clone has survived for millennia, enduring climate extremes, under a strategy of rapid growth to escape browsing as a physiological trade-off for low resistance [37–38]. However, we acknowledge that fluctuations in herbivory have likely taken place over such a time span. This rapid growth hypothesis corresponds well with a molecular comparison of diploid and triploid aspen growth as measured in tree-ring growth in and around Pando [24]. In brief, DeRose et al. [24] showed that growth rates overall, but particularly in early ramet development, were faster in triploids including the Pando clone. In terms of the resistance *vs*. escape [39] formula at Pando we are left to conclude that a disruption to this balance has taken place regarding consumptive patterns of ungulates. Where ungulate numbers likely ebbed on annual time scales, the current dearth of recruitment suggests a decadal imbalance threatening survival of a very long lived clone. We must begin to fully understand this relatively simple (genetically) aspen forest if we are to be able to tackle complex aspen landscapes with myriad variations in genetic composition and herbivore pressures.

Qualitative evidence in the form of the aerial photo record of recent decades (Fig 3) presents a putative explanation for novel browse patterns at Pando. We know that historic removal of ungulate predators has shaped aspen forests even though the level of that impact is disputed [14,39]. Extrication of wild predators and prohibitions on hunting near recreation sites facilitated a “refuge affect” for ungulates that are accustomed to people (mule deer, cattle) while discouraging those that are not (Rocky Mountain elk). This altered pattern roughly coincides with our 72-year photo sequence, when increases in road traffic, recreational home development, and campground use have flourished. Moreover, while most aspen stems do not live past 100–130 years, the current mature cohort is near that limit now; the difference is that in 1939 there were ample recruitment trees existing in a complex vertical forest structure to continually replace dying overstory. Loss of that resilient stand architecture in tandem with present-day browsing patterns and inadequate protection provides a potential explanation for Pando’s threatened status. Further testing of these ideas presents a logical course for future investigation which we hope will lead to multi-disciplined solutions for ecological recovery.

### Implications for mega-conservation

This story, minus the single-genotype novelty, is being repeated at very large scales where aspen forests—predominantly *P*. *tremula* and *P*. *tremuloides*, but other upland *Populus* species known as “aspen,” too—support regionally disproportionate biodiversity levels [1–5]. Where continental-scale conservation commonly focuses on small populations of species in narrow habitat niches, an alternative strategy which aspires to greater overall species preservation may take aim at widely spread species-rich systems facing common deleterious agents (i.e., increased drought, elevated herbivory, fire suppression, development and type conversion).

In particular, warming regional climates are already leading to incidents of reduced growth in aspen [40–42]. At Pando, monotypic gene composition may help us understand aspen’s response to changing environmental conditions and human impacts so that we may apply that knowledge to more complex aspen landscapes regionally. For example, if we can understand how a single genotype forest responds to chronic herbivory, then we have a building block in place to begin examining more complex aspen landscapes where herbivory places a central role—a phenomenon that appears to be widespread (e.g., [3,11] and genetics-based chemical defense is an underexplored aspect of this problem [36,37]. Warming and drying climates alone may not be enough to reduce aspen cover; while these factors may reduce the “realized niche” [43,44] of aspen they may also increase wildfire which promotes aspen rejuvenation [7,45]. However, when coupling increased drought with unchecked herbivory, particularly in stable aspen types, such communities are subject to elevated risk of collapse [8]. Cascading effects of increased temperatures and reduced snow cover (i.e., lengthening “browse season”) have also been shown to impact aspen-dependent birds, with the overall effect of decreasing biodiversity [9]. Numerous studies in both European and North American aspen have documented the keystone nature (i.e., many species reliant on one species) of these forests for a host of plant and animal functional groups [3,5–6,46–49]. Such keystone aspen systems are at risk across global-scales where both *P*. *tremula* and *P*. *tremuloides* have been threatened by widespread effects of ungulate herbivory [3,8,12,14–16,48,50–51]. Therefore, deterioration of aspen forests, whether locally or continentally, carries great potential to amplify biodiversity losses. While degradation of aspen—currently occurring at Pando—is often affected by multiple factors, a common theme of overabundant ungulates compounded by stressors associated with changing climates, should be expected for wide areas of the northern hemisphere. Having said this, we recognize regional fluctuations—whether natural or policy-driven—such as fire suppression, size, and intensity, as well as presence of apex predators affecting ungulate browse patterns [3,12,45,52], constitute important factors not addressed in the present study. Thus, collaboration across disciplinary lines will be required to ensure the greatest resilience in the face of this change. To do otherwise may endanger broad geographic swaths of ecological integrity and biodiversity.

## Conclusion

This first comprehensive assessment of conditions at the famed Pando aspen clone reveals an ancient forest threatened by recent human decisions. A vital lesson derived from this study is that independently managing vegetation and wildlife may harm both. While several human alterations to this forest have taken place in recent decades, it is the lack of simultaneous herbivore regulation that has caused this stand’s degeneration. Here and previously [23] we have documented some successes with experimental treatments combined with post-treatment browse protection that show promise for Pando’s outlook. Through these tests and our aerial photo documentation of 1987–88 failed cutting experiments, it is clear that both passive and active management cannot be successful without mitigating herbivory. Positive results within the 2013 fence unfortunately contrast with continuing recruitment failures outside the fence, as well as within the 2014 fence. The majority of this giant clone remains either insufficiently protected via a penetrable fence or totally unguarded from herbivory; both instances are expected to yield a greatly reduced clone and cascading effects to obligate species [1,53–55]. Moreover, we highlight that post-disturbance stable-type aspen forests such as Pando require a complex vertical stand structure for system resilience [13,31–32]. Even where a 25-year-old clearfell and fence yielded advanced recruitment today, no successful reproduction since that time presents significant concerns. Greater vigilance will be required in future forest-herbivory management via deer and cattle exclusion, increased animal movement, or population reductions if we wish to see a rebounding and resilient Pando clone.

This spatially confined study on a genetically uniform aspen forest in Utah has broad ramifications for aspen management at-large; the issues found here are mirrored across the ranges of the most widespread tree species in the world (i.e., *P*. *tremuloides*, *P*. *tremula*; [3,48–49,55–56]). As we begin to understand growth and defense mechanisms linked to genotype, there is potential for altering aspen-herbivore strategies accordingly. We may use this relatively simple system, which is still an entire forest, as a building block for understanding and mitigation of herbivore impacts at larger scales in multiple-clone landscapes with greater diversity of defense chemistry allocation. Likewise, experimental tradeoffs between nutrition, defense, growth, and response to disturbance may be compared to this iconic clone as a starting point for understanding long-term survival of aspen communities under duress.

While conservation efforts are commonly aimed at rare species within specialized niches, a habitat approach affecting a broad array of aspen-obligates at continental-scales may be a more effective strategy for preserving greater overall biotic integrity. Impacts of overabundant wild ungulates are often overlooked by preservationists, policymakers, and the public because of their perceived benign effects [56]. What many conservation efforts have failed to implement thus far are coordinated strategies of forest-herbivore stewardship, particularly at regional and multinational scales [3,13,57]. Such approaches not only promote ecological, process-based, landscape management, they have the added benefit of clearly linking human decision-making to cascading ecological consequences. There should be no confusion over the point that both domestic and wild herbivore populations are governed by people’s preferences and actions, and that those decisions result in long-term consequences for ecosystems writ large.

## Supporting information

S1 Table Master data set for all field plots used in this study. (PDF)
