# Supplementary material for: Mule deer impede Pando’s recovery: Implications for aspen resilience from a single-genotype forest
Source: PLoS One. 2018 Oct 17;13(10):e0203619. doi: 10.1371/journal.pone.0203619 (PMC6192553; doi:10.1371/journal.pone.0203619)
Supplement: S1 Table — (PDF) [file pone.0203619.s001.pdf]

Pando Monitoring master data set Phases 1 & II - 2017 - 65 plots total (1-33 Phase 1; 102-218 Phase II)

Plot#	UTM_E	UTM_N	Elev	#layers	Stand cond	Treatment	Protection (none, lower, upper)	Common Juniper Cover (%)	Aspen Cover (%)
1	434786	4264125	2701	1	3	1	1	5	20
2	434955	4264574	2706	1	3	1	1	5	17
3	434864	4264270	2697	1	3	1	1	0	0
5	434612	4264094	2706	1	3	1	1	8	13
6	434712	4264028	2702	2	3	1	1	18	51
7	434564	4264163	2717	2	2	1	1	8	37
8	434853	4264457	2711	2	2	5	2	45	19
9	434896	4264437	2705	2	2	5	2	5	34
12	434769	4264349	2713	1	3	4	2	15	10
13	434805	4264353	2708	1	3	4	2	3	21
14	434716	4264203	2706	2	3	3	2	20	11
15	434726	4264248	2709	2	3	3	2	65	22
16	434747	4264233	2702	2	2	3	2	35	17
17	434704	4264163	2704	1	3	6	2	20	13
18	434660	4264225	2718	1	3	6	2	50	18
19	434663	4264183	2707	1	3	6	2	45	31
20	434774	4264268	2705	2	3	7	2	10	15
21	434742	4264282	2709	2	3	7	2	15	27
22	434763	4264295	2710	2	2	7	2	35	24
24	434842	4264389	2709	1	3	8	2	10	20
25	434828	4264437	2702	2	3	8	2	8	18
26	434709	4264133	2702	2	3	2	2	20	14
27	434618	4264196	2711	2	3	2	2	30	14
29	434701	4264266	2718	2	3	2	2	38	18
30	434846	4264347	2704	1	3	2	2	5	20
32	434846	4264497	2711	2	2	2	2	15	0
33	434921	4264497	2710	1	2	2	2	26	4
102	434498	4264190	2727	1	3	10	3	18	35
105	434498	4264240	2734	1	3	10	3	35	21
106	434548	4264240	2728	1	3	10	3	55	44
109	434448	4264290	2738	1	3	9	3	30	0
111	434548	4264290	2730	1	3	10	3	15	23
116	434448	4264340	2747	1	3	9	3	35	3
117	434498	4264340	2741	1	3	9	3	45	13
120	434648	4264340	2720	2	3	10	3	43	43
123	434448	4264390	2759	1	3	9	3	45	21
124	434498	4264390	2758	1	3	9	3	60	29
126	434598	4264390	2729	1	3	9	3	25	11
129	434348	4264440	2788	1	3	9	3	20	43
131	434448	4264440	2768	1	3	9	3	25	39
132	434498	4264440	2770	1	3	9	3	45	33

134	434598	4264440	2740	1	3	9	3	40	31
136	434698	4264440	2727	2	3	10	3	20	40
139	434498	4264490	2766	1	3	9	3	35	38
142	434648	4264490	2744	1	3	9	3	65	17
145	434448	4264540	2775	1	3	9	3	33	28
150	434698	4264540	2730	1	3	9	3	6	32
156	434598	4264590	2757	1	3	9	3	63	49
157	434648	4264590	2748	1	3	9	3	20	32
162	434548	4264640	2769	1	3	1	1	25	34
165	434798	4264640	2732	1	3	1	1	35	19
167	434498	4264690	2769	1	3	1	1	6	40
169	434598	4264690	2738	1	3	1	1	63	35
171	434748	4264690	2735	1	3	1	1	73	33
173	434848	4264690	2727	1	3	1	1	24	23
176	434848	4264740	2741	1	3	1	1	2	21
177	434898	4264740	2723	1	3	1	1	7	24
180	434798	4264790	2735	1	3	1	1	18	19
183	434948	4264790	2728	1	3	1	1	18	19
201	434448	4263940	2710	1	3	1	1	15	15
203	434548	4263940	2710	1	3	1	1	12	20
207	434498	4264040	2717	1	3	1	1	60	18
211	434398	4264040	2720	1	3	1	1	4	19
215	434598	4264090	2710	2	3	1	1	43	23
218	434548	4264090	2713	1	3	1	1	40	13

from 2016

Regen. Count	Regen./ha	Regen. Browse%	Recruit. Count	Recruit/ha	Tree count	tph	Live tree count	live tph	live BA
1	83	100	0	0	3	250	3	250	14
2	167	100	0	0	6	500	6	500	17
3	250	100	0	0	0	0	0	0	0
0	0	0	0	0	1	83	1	83	6
3	250	100	0	0	23	1917	22	1833	59
3	250	100	0	0	11	917	10	833	34
4	333	0	0	0	5	417	3	250	13
17	1417	0	1	83	13	1083	9	750	15
12	1000	0	0	0	4	333	3	250	8
14	1167	0	1	83	2	167	2	167	7
36	3000	8	3	250	4	333	2	167	7
14	1167	0	0	0	9	750	6	500	15
12	1000	0	1	83	5	417	3	250	13
24	2000	0	1	83	4	333	4	333	20
33	2750	0	1	83	3	250	3	250	18
61	5083	0	2	167	6	500	5	417	19
18	1500	0	1	83	8	667	5	417	5
23	1917	0	0	0	4	333	2	167	2
26	2167	0	0	0	7	583	6	500	15
21	1750	0	0	0	3	250	2	167	18
10	833	0	0	0	9	750	5	417	13
11	917	0	0	0	1	83	1	83	3
9	750	0	1	83	3	250	2	167	12
27	2250	0	1	83	5	417	4	333	8
10	833	0	0	0	1	83	1	83	3
12	1000	0	2	167	4	333	4	333	3
34	2833	0	0	0	13	1083	10	833	10
0	0	0	45	3750	14	1167	14	1167	12
2	167	50	47	3917	2	167	2	167	2
0	0	0	77	6801	11	917	11	917	10
0	0	0	0	0	0	0	0	0	0
0	0	0	14	1237	2	167	2	167	2
9	750	33	0	0	0	0	0	0	0
0	0	0	0	0	3	250	1	83	6
0	0	0	77	6801	13	1083	13	1083	11
1	83	100	0	0	2	167	1	83	6
1	83	0	0	0	10	833	7	583	22
4	333	25	0	0	2	167	1	83	3
5	417	60	0	0	9	750	9	750	30
2	167	100	0	0	6	500	4	333	3
0	0	0	0	0	8	667	6	500	22

0	0	0	0	0	6	500	4	333	17
0	0	0	45	3975	18	1500	18	1500	16
0	0	0	0	0	7	583	5	417	23
1	83	100	0	0	2	167	2	167	9
3	250	33	0	0	4	333	3	250	9
12	1000	25	0	0	3	250	3	250	14
0	0	0	0	0	13	1083	9	750	43
0	0	0	0	0	11	917	9	750	43
0	0	0	1	88	6	500	5	417	17
0	0	0	0	0	6	500	3	250	11
0	0	0	0	0	15	1250	12	1000	38
0	0	0	0	0	6	500	6	500	19
0	0	0	0	0	8	667	7	583	35
0	0	0	0	0	7	583	6	500	22
13	1083	15	0	0	1	83	1	83	6
4	333	25	0	0	2	167	2	167	12
1	83	100	0	0	1	83	1	83	3
1	83	100	0	0	6	500	4	333	24
5	417	40	0	0	6	500	4	333	10
2	167	50	1	88	3	250	3	250	3
1	83	100	0	0	4	333	3	250	18
1	83	100	0	0	3	250	3	250	6
38	3167	100	1	88	7	583	6	500	24
1	83	100	1	88	4	333	2	167	9

dead BA	dead BA as % of Total BA	total BA	Cattle scat/ha	Deer scat/ha
0	0	14	83	0
0	0	17	0	333
0	0	0	1167	0
0	0	6	1417	750
6	9	65	667	83
6	15	40	83	0
7	35	19	0	0
3	19	18	0	0
6	44	14	0	0
0	0	7	0	0
12	64	19	0	0
3	15	18	0	0
2	12	14	0	0
0	0	20	0	0
0	0	18	0	0
1	4	20	0	0
4	45	10	0	0
2	50	3	0	0
6	28	21	0	0
0	0	18	0	0
5	29	18	0	0
0	0	3	0	0
6	33	18	0	0
6	41	14	0	0
0	0	3	0	0
0	0	3	0	0
3	20	13	0	0
0	0	12	0	0
0	0	2	0	83
0	0	10	0	0
0	0	0	0	333
0	0	2	0	417
0	0	0	0	500
9	59	14	0	0
0	0	11	0	0
6	50	12	0	83
6	22	28	0	0
3	50	5	0	0
0	0	30	0	0
7	66	10	0	167
3	14	26	0	83

9	33	26	0	0
0	0	16	0	0
9	27	31	0	0
0	0	9	0	83
3	22	12	0	83
0	0	14	0	583
17	28	60	0	167
9	16	52	0	0
6	26	22	0	750
11	50	22	0	0
8	17	46	0	333
0	0	19	0	167
3	7	37	0	417
6	21	28	0	0
0	0	6	0	0
0	0	12	0	250
0	0	3	0	0
12	33	35	0	0
5	33	16	0	0
0	0	3	0	167
3	13	20	0	250
0	0	6	0	0
1	4	25	0	0
9	50	17	0	0
